# Metagenomic Analysis Identified *Stenotrophomonas maltophilia* Pneumonia in an Infant Suffering From Unexplained Very Severe Pneumonia

**DOI:** 10.3389/fped.2019.00380

**Published:** 2019-09-19

**Authors:** Yan Lin, Bao-Xi Wang, Ni-Ni Zhang, Lei Zhang, Zhi-Bo Gao, Jiao Tian, Xun Jiang

**Affiliations:** Department of Pediatrics, Tangdu Hospital, Air Force Medical University, Xi'an, China

**Keywords:** severe pneumonia, *Stenotrophomonas maltophilia*, infant, next-generation sequencing, metagenomics

## Abstract

Pneumonia poses a significant global morbidity and mortality burden on children. Etiological diagnosis and matched anti-microbial therapy are particularly important for very severe pneumonia. Although great advances have been achieved in diagnostic approaches, it remains challenging to identify pathogens in unexplained pneumonia (UP) cases. In this study, we applied next-generation sequencing (NGS) technology and a metagenomic approach to detect and characterize respiratory bactiera in an UP case in infant. *Stenotrophomonas maltophilia* was the only bacterial pathogen detected in blood. Metagenomic sequencing also provided bacteria genomic sequences, which could be used to evaluate the role of this pathogen in the disease. This NGS method has the potential to improve the identification of causative organisms in patients with pneumonia and the delivery of appropriate, pathogen-directed antibiotic therapy.

## Introduction

Globally, pneumonia is the top cause of death in children with age standardized mortality rates of 38.9/100,000 children. Furthermore, pneumonia remained one of the leading causes of disability-adjusted life years in children (1, 2). The World Health Organization (WHO) guideline defines “very severe pneumonia” as children with tachypnea and general danger signs (e.g., severe chest in-drawing, cyanosis, difficulty breastfeeding, unconsciousness) (3). With the development of detecting techniques, we have more detection methods to identify pathogens (e.g., immunofluorescence technique or polymerase chain reaction of respiratory secretion), comparing with traditional respiratory secretion culture and blood culture. But it remains challenging to identify pathogens in unexplained pneumonia (UP) cases. We present here a case of *Stenotrophomonas maltophilia* severe pneumonia in an infant diagnosed by Next-generation sequencing method. Furthermore, metagenomic sequencing afforded information regarding the quantity of each pathogens in the sample, which could be used to evaluate the role of each pathogen in the disease. Our study highlights the potential of metagenomic sequencing for pathogen identification in UP cases.

## Case Report

A 10-week-old extremely preterm girl was admitted to our hospital for severe paroxysmal cyanosis and tachypnea. The infant was the product of a 25-week-and-4-day gestation and was a test-tube baby, from a gestational diabetes mother. The pregnancy was otherwise uneventful. A vaginal delivery was performed. The infant was 890 g with Apgar scores of 5, 9, and 9 at 1, 5, and 10 min, respectively. The infant was independent on the oxygen when she was discharged after 58 days of hospitalization with 2,020 g.

**Figure 1:**
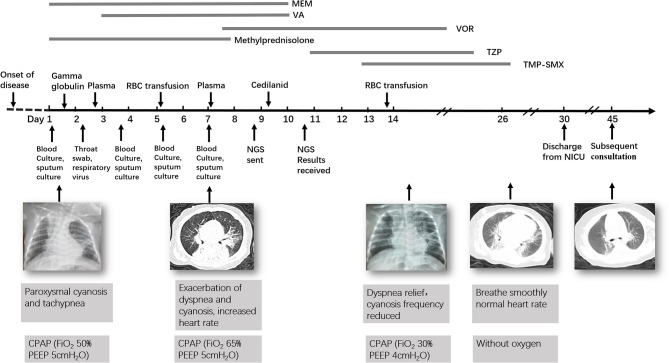
Timeline of the patient's clinical course and outcome. MEM, Meropenem; VA, Vancomycin; VOR, Voriconazole; TZP, Tazobactam and Piperacillin; TMP-SMX, Trimethoprim/Sulfamethoxazole.

**Figure 2:**
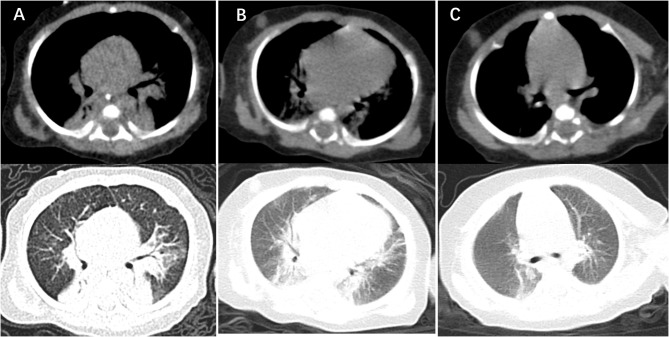
Computer tomography imaging showing lung lesion. **(A)** CT of the chest obtained around the time of clinical presentation most severely, showing bilateral alveolar multiple patchy shadows and local consolidation, more prominent on the left lung than right. **(B)** CT of the chest obtained after treatment 14 days with TMP-SMX, showing absorption multiple patchy and local consolidation. **(C)** CT of the chest obtained around the time of 2 weeks after discharge from NICU, showing that dual markings slightly increased, the lung parenchyma was no significant consolidation.

At day of life 70, the infant presented with symptoms of poor response and appetite, but her parents did not notice these symptoms. At day of life 72, the infant has symptoms of paroxysmal cyanosis and tachypnea, then was subsequently admitted to our hospital. Upon arrival to our hospital, vital signs were notable for respiratory rate of 70 per minute and heart rate of 170 beats per minute. Physical exam revealed coarse breath sounds and pulmonary moist rales. The infant was diagnosed with severe pneumonia and we immediately gave CPAP assisted ventilation (FiO_2_ 50% PEEP 5 cmH_2_O). Meanwhile, we actively carried out blood culture, sputum culture, throat swab, respiratory virus (respiratory syncytial virus, influenza viruses, parainfluenza viruses, adenovirus, rhinovirus) and mycoplasma detection from blood and galactomannan detection. Then intravenous fluids and meropenem were administered (Figure 1). Chest X-ray showed high-density shadow in the left middle lobe lung on Day 1. But her general condition progressively worsened after 3 days. Her white blood cell count increased from 5.71 × 10^9^/L (with 52% neutrophils and 27.5% lymphocytes) to 16.37 × 10^9^/L (with 74.9% neutrophils and 16.7% lymphocytes), whereas her C-reactive protein and procalcitonin levels were normal. Pulmonary atelectasis in the right upper lobe and bilateral alveolar consolidation by chest computed tomography on Day 6 after the onset of symptoms (Figure 2A). Meanwhile repeated blood and sputum secretions were negative for bacteria, respiratory virus, tuberculosis, and fungi in cultures, and G-test (1,3 -beta-D-glucan test) and GM-test (galactomannan test), respectively. Her oxygenation index was 125 at admission, indicating a bad oxygenation status. And her echocardiography and blood pro-brain natriuretic peptide readings were normal. During this period, the infant showed polypnea, paroxysmal dysphoria, cough, and abundant yellow sticky respiratory secretions. Given the concern for a severe infection, empiric broad-spectrum antibiotics consisting of meropenem, vancomycin, and voriconazole to cover typical bacterial pathogens and fungal infection after a second infectious workup was performed including repeated blood culture, sputum culture (Figure 1).

**Figure 3:**
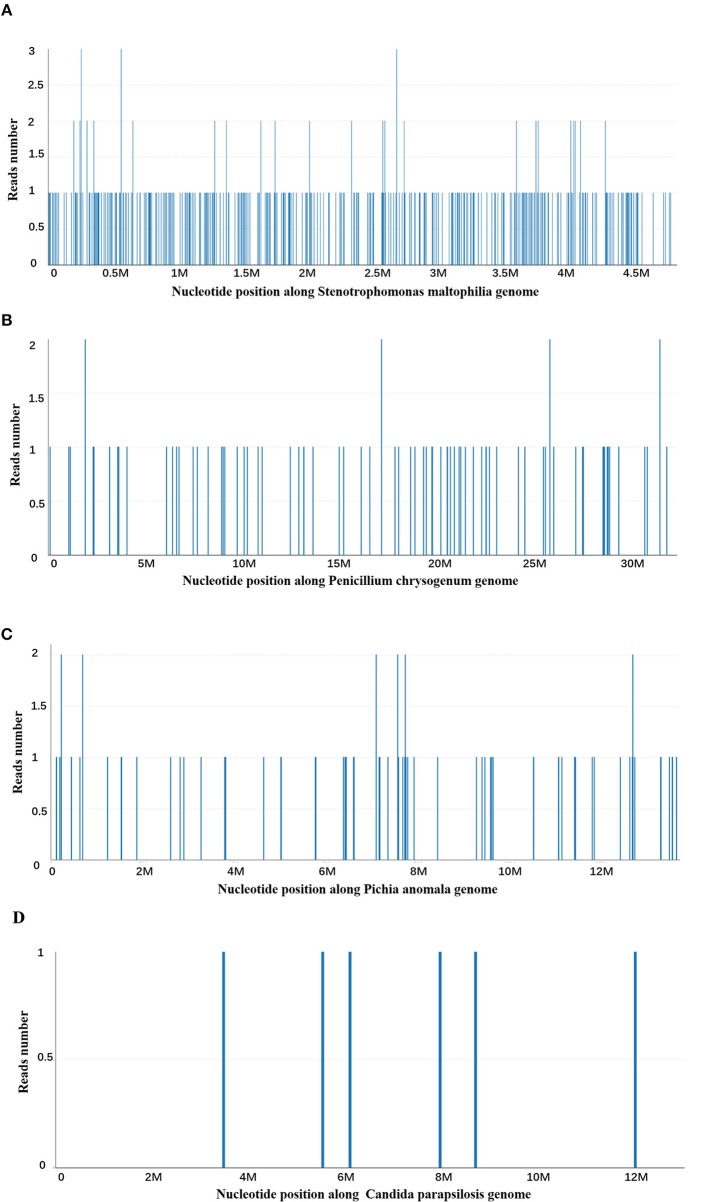
Next-generation sequencing and a metagenomic analysis. **(A)** *Stenotrophomonas maltophilia* specific reads and its nucleotide position along *Stenotrophomonas maltophilia* genome. **(B)** *Penicillium chrysogenum* specific reads and its nucleotide position along *Penicillium chrysogenum* genome. **(C)** *Pichia anomala* specific reads and its nucleotide position along *Pichia anomala* genome. **(D)** *Candida parapsilosis* specific reads and its nucleotide position along *Candida parapsilosis* genome.

At day 10, the microbial species were then assessed by metagenomic analysis. Deoxyribonucleic acid libraries for Next-generation sequencing (NGS) were prepared as previously described (4, 5). Sequencing was performed on an Illumina MiSeq instrument. Total numbers of raw reads were 13849671, and non-human reads accounted for 5.92%. *Stenotrophomonas maltophilia* reads (428) were the only bacterial pathogen in blood (Figure 3A). In addition, we also detected several fungi pathogens in blood, including *Penicillium chrysogenum* reads (404), *Pichia anomala* reads (134), and *Candida parapsilosis* reads (6) (Figures 3B–D). The infant was then treated with Tazobactam and Piperacillin (TZP) and trimethoprim/sulfamethoxazole (TMP-SMX) successively, subjected to non-invasive positive pressure ventilation. Her oxygenation status and chest computed tomography showed significant improvement after treatments (Figure 2B), and the infant was discharged on Day 30 after admission. Two weeks after discharge, the infant appeared good responses and appetite, and her chest computed tomography showed distribution of the two slightly thickened lung markings, clear lung fields and no substantive changes (Figure 2C).

## Discussion

Bacteria, virus, and fungi are the major pathogens of human respiratory infections. However, pathogens remain undetected by routine clinical testing in ~30% of respiratory cases (6). Therefore, for these patients, we need to choose more sensitive and effective ways to detect pathogens for etiological diagnosis and reasonable treatment. In this study, we report a case of unexplained very severe pneumonia due to *S. maltophilia* that was diagnosed by plasma whole-genome NGS method.

*Stenotrophomonas maltophilia* is a commensal and an emerging pathogen earlier noted in broad-spectrum life-threatening infections among the vulnerable, but more recently as a pathogen in immunocompetent individuals (7). In addition, *S. maltophilia* has emerged as an important pathogen that induces nosocomial infections (8). *S. maltophilia* is a non-fermentative, gram-negative bacillus and causes severe infectious diseases, such as pneumonia, bacteremia, skin and soft-tissue infection, urinary tract infection, and meningitis (9–12). In recent times, the consistency of infection by the organism as reported worldwide is quite alarming. *S. maltophilia* accounts for about 3.7% (*n* = 10,000) in hospital discharges (13–15). A recovery rate of 3.3% in *S. maltophilia* infections was reported in the United States (16). And the mortality rate due to *S. maltophilia* infection was 36.6% rate found in a large scale study (17) and 25–51% in a multi-center study (18). Pathogenesis is by colonization, rather than infection, which is often accompanied by tissue invasion (19). The most important risk factors for *S. maltophilia* infection in neonates and infants are invasive procedures; previous exposure to antibiotics, such as carbapenem and aminoglycoside; and prolonged hospitalization (20). The duration of hospitalization of some patients before the onset of the Stenotrophomonas related clinical features and/or diagnosis is an important factor in nosocomial infection. Exemplifying case studies considered the duration of hospitalization before the onset of *S. maltophilia* bacteremia, which ranged from 11.5 to 24 days (21, 22). Neonate and infants have low immunity, and often have severe and uncontrollable symptoms after infection. *S. maltophilia* is intrinsically resistant to lots of antibiotics, including carbapenem and aminoglycosides which are used empirically for nosocomial infection (8). Therefore, early identification and appropriate treatment are important.

In principle, conventional cultural methods on nutrient agar support the growth of *S. maltophilia*, although certain strains require methionine (23, 24). However, we did not find any other pathogenic bacteria in conventional blood culture and sputum culture, which brought great difficulties to diagnosis and treatment. The reason may be inappropriate culture media or low bacterial counts but high virulent. We treated the children with empirical broad-spectrum antibiotics but her symptoms gradually worsened. Therefore, we choose microbial sequencing research to further explore pathogens. Common methods include 16S rDNA sequencing and metagenomic sequencing. Metagenomic sequencing has great potential utility in the diagnosis of infectious disease since it can obtain all kinds of pathogen information, including bacteria, viruses, fungi, and parasites, etc., and simultaneously interrogate host responses. While 16S rDNA refers to the identification of bacteria species by using the method of 16S rDNA sequence, which can only detect bacteria-like pathogens and that cannot fully cover the source of infection in clinical samples. In addition, metagenomic sequencing has multiple advantages compared with the 16S rDNA sequencing method including increased detection of diversity, increased prediction of genes and improved the accuracy of species detection (25). In this case, we did not find any other pathogens in repeated blood culture, sputum culture and other etiological examination, and her C-reactive protein and procalcitonin levels were normal during whole clinical course of the pneumonia. Therefore, we need total metagenomics to cover a broader range of pathogens.

In this study, we applied next-generation sequencing (NGS) technology and a metagenomic approach to detect *S. maltophilia* infection in infant. Metagenomics provides a means of assessing the total genetic pool of all the microbes in a sample, in a culture-independent manner (26). Firstly, it only takes 48 h to have the identification, which is faster than the traditional bacterial culture. Secondly, these approaches have the benefit of increasing the number and proportion of pathogen reads in the sequence data, which can increase the detection sensitivity for microorganisms (26–28). In addition, key advantage of NGS approaches is that the sequencing data can potentially be leveraged or additional analyzed beyond the mere identification of a causative pathogen, such as microbiome characterization and parallel analyses of human host responses through transcriptome profiling by RNA sequencing (RNA-seq). Thus, the clinical utility of NGS in diagnosis may be in the most difficult-to-diagnose cases or for immunocompromised patients, in whom the spectrum of potential pathogens is greater (29). In principle, the sensitivity, specificity and credibility of the body fluids or tissue samples from the infected site are higher, such as bronchoalveolar lavage fluid (BALF) for pneumonia. However, due to the young age and serious condition of the infant, BALF was not suitable, we selected blood samples for testing. Due to the abundant blood vessels in the lungs of small infants, pathogens or toxins produced by them can enter the blood, which makes the detection rate of pathogens in the blood significantly increased. NGS combines molecular biology techniques and informatics to filter human sequences and identify any microorganism sequences directly from patient plasma (30). Moreover, metagenomic sequencing could provide detailed genomic information of the pathogens presented in samples, which could be used for genotype characterization and phylogenetic analysis (31).

However, a positive pathogen test is not always meaning an infectious pathogen. To determine whether it is contaminated bacteria, colonizing bacteria or pathogenic bacteria, it requires a comprehensive analysis according to the infection site, clinical manifestations of patients, characteristics of medical history, environmental conditions, immune status, imaging, hematological examination, and response to empirical treatment. In this case, the peripherally inserted central venous catheters (PICC) was established 1 week after birth, and the PICC was removed on the day before her first discharge, which was used for 50 days. After the first admission, the infant was treated with meropenem, vancomycin, and cefoperazone sodium sulbactam successively, and the final exposure to broad-spectrum antibiotics was at 18 days before first discharge with cefoperazone sodium and sulbactam sodium. Infants, particularly those born prematurely, receive extensive antibiotic therapy during the first weeks of life may have *S. maltophilia* colonization in the respiratory tract (20). The infant was the extremely preterm of a 25-week-and-4-day gestation and had the most important risk factors for *S. maltophilia* infection, such as PICC, previous exposure to antibiotics in her early life and prolonged hospitalization. Futhermore, clinical manifestations of this infant and responses to empirical treatment were consistent with the characteristics of *S. maltophilia* infection. Therefore, we selected the antibiotic, TZP and TMP/SMX, which are sensitive to *S. maltophilia*. In addition, we also detected several fungal pathogens in blood, including *Penicillium chrysogenum* reads (404), *Pichia anomala* reads (134), and *Candida parapsilosis* reads (6). Due to the negative fungal tests (G-test, GM-test, and Fungal culture) and her clinical manifestations, we did not consider that this infant suffered severe fungal infection. Furthermore, the infant was prophylactic treated with VOR that can cover these fungal pathogens before NGS results received.

After receiving the NGS results, we firstly considered stopping meropenem and vancomycin because of their natural resistance to *S. maltophilia*. In addition, we cannot confirm the infection of *S. maltophilia* or the possibility of contamination, so we chose broad-spectrum antibiotics, TZP, that *S. maltophilia* were sensitive to at the same time. But there was no significant improvement in symptoms after 2 days' treatment in this case. Previous studies have shown that TZP can be a candidate for combination therapy in treating *S. maltophilia* because isolates showed a high susceptibility rate to this combination. However, TZP are not suitable for monotherapeutic strategy for treating *S. maltophilia* because this microorganism has a high intrinsic resistance to most penicillins and cephalosporins, as well as to all carbapenems (32). Thus, we added TMP-SMX combined treatment considering the severity of the disease and the possible insensitivity to TZP. TMP/SMX, ticarcillin-clavulanate, fluoroquinolone, colistin, and tigecycline are candidates that show consistent therapeutic activity against *S. maltophilia*. Although increasing resistance of *S. maltophilia* to TMP-SMX has been reported (8), the present study suggests that TMP-SMX may still be suitable as first-line treatment (7), and be recommended for being most potent antibiotics against *S. maltophilia* (22). In addition, TMP-SMX has been recommended by a number of researchers as initial therapeutic option for serious *S. maltophilia* infection (33). Wang et al. showed that clinical success rates of monotherapy with TMP-SMX was 61% (34). In this case, we have treated infant with TMP/SMX showing benefit outcomes. Studies showed that treatment of *S. maltophilia* infection with a combination of two or three antimicrobials can be considered in the current practice because *S. maltophilia* has a high resistance rate (34, 35). And in this case, it may be the synergistic effects of the two antibiotics.

In conclusion, *S. maltophilia* should be considered as a possible infectious agent in pediatric patients with high Stenotrophomonas infection risk factors. For unexplained very severe pneumonia, NGS will enhance the diagnostic rate for “unknown” pathogens in pediatrics and facilitate the identification of new pathogens. Ongoing technological and bioinformatic innovations, such as the NGS diagnostic tools, will hopefully apply to the clinic for timely, targeted, and precise antibiotic use in the ICU.

## Data Availability Statement

The raw data can be accessed from EBI metagenomics (Accession: PRJEB32856).

## Ethics Statement

Written informed consent was obtained from the individual(s) and minor(s)' legal guardian/next of kin, for the publication of any potentially identifiable images or data included in this article.

## Author Contributions

YL, B-XW, and XJ conceptualized and designed the study, drafted the initial manuscript, and reviewed. YL and N-NZ reviewed and revised the manuscript. YL, LZ, Z-BG, and JT designed the data collection instruments, collected data, carried out the initial analyses. All authors approved the final manuscript as submitted and agree to be accountable for all aspects of the work.

### Conflict of Interest

The authors declare that the research was conducted in the absence of any commercial or financial relationships that could be construed as a potential conflict of interest.
